# Portal hypertensive enteropathy diagnosed by capsule endoscopy and demonstration of the ileal changes after transjugular intrahepatic portosystemic shunt placement: a case report

**DOI:** 10.1186/1752-1947-5-90

**Published:** 2011-03-04

**Authors:** Alessandro Pezzoli, Nadia Fusetti, Loredana Simone, Angelo Zelante, Viviana Cifalà, Alessandra Carella, Sergio Gullini

**Affiliations:** 1 Department of Gastroenterology and GI Endoscopy, Arcispedale S.Anna, Corso Giovecca 203, 44100 Ferrara, Italy

## Abstract

**Introduction:**

Recent data suggest that mucosal abnormalities can occur even in the duodenum, jejunum, and distal ileum of cirrhosis patients. We present a case of portal hypertensive enteropathy in a cirrhosis patient shown by capsule endoscopy and the effect of transjugular intrahepatic portosystemic shunt on the ileal pictures.

**Case presentation:**

An 83-year-old Caucasian woman was admitted to our hospital for anemia and a positive fecal occult blood test. An upper gastrointestinal endoscopy revealed small varices without bleeding signs and hypertensive gastropathy. Colonoscopy was negative. To rule out any other cause of bleeding, capsule endoscopy was performed; capsule endoscopy revealed severe hyperemia of the jejunum-ileal mucosa with active bleeding. Because of the persistence of anemia and the frequent blood transfusions, not responding to β-blocker drugs or octreotide infusion, a transjugular intrahepatic portosystemic shunt was performed. Anemia improved quickly after the transjugular intrahepatic portosystemic shunt, and no further blood transfusion was necessary in the follow-up. The patient developed portal encephalopathy two months later and was readmitted to our department. We repeated the capsule endoscopy that showed a significant improvement of the gastric and ileal mucosa without any signs of bleeding.

**Conclusion:**

Hypertensive enteropathy is a rare condition, but it seems more common with the introduction of capsule endoscopy in clinical practice. This case shows that the jejunum can be a source of bleeding in cirrhosis patients, and this is the first demonstration of its resolution after transjugular intrahepatic portosystemic shunt placement.

## Introduction

Changes in the gastric mucosa are a well-known aspect of cirrhosis in patients with portal hypertension [1]. Some data suggest that similar abnormalities can also occur in the duodenum, jejunum, and distal ileum [2-4]. With the introduction of capsule endoscopy (CE) in clinical practice, these changes can be detected easily, and the so-called "portal hypertensive enteropathy" [5] seems more common [6-9]. We present a case of portal hypertensive enteropathy (PHE) in a cirrhosis patient shown by CE and the effect of transjugular intrahepatic portosystemic shunt (TIPS) placement on the ileal pictures.

## Case presentation

**Figure 1:**
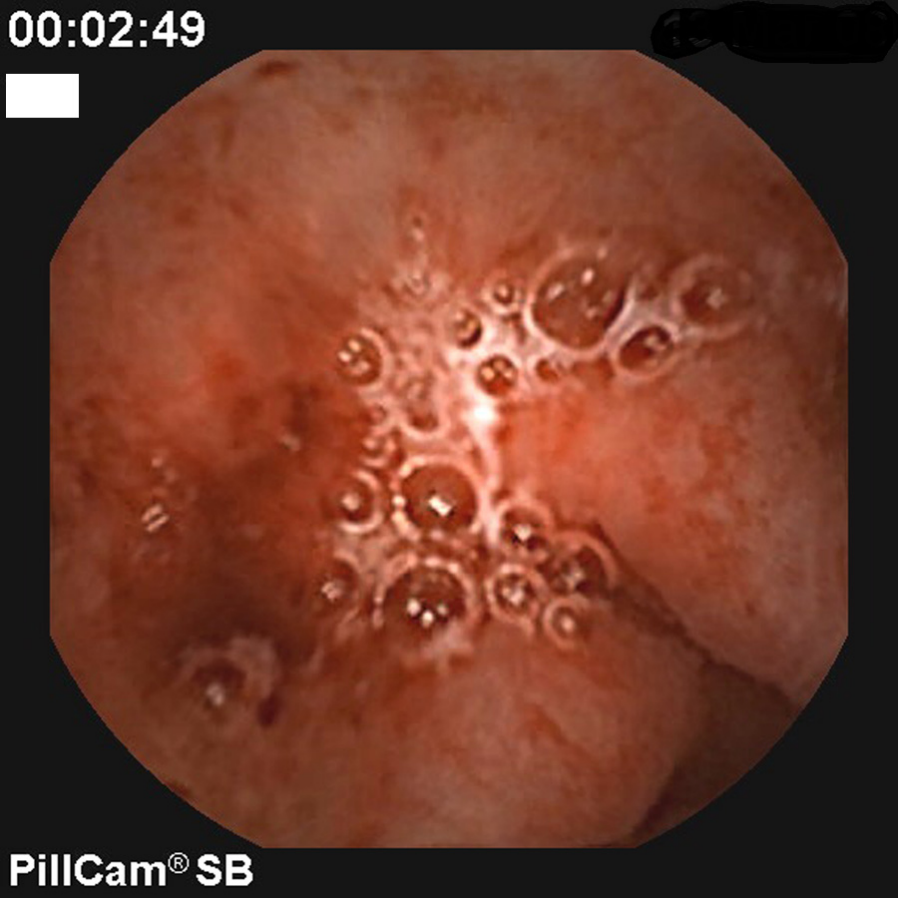
**Portal hypertensive gastropathy: severe portal hypertensive gastropathy with minimal oozing**.

**Figure 2:**
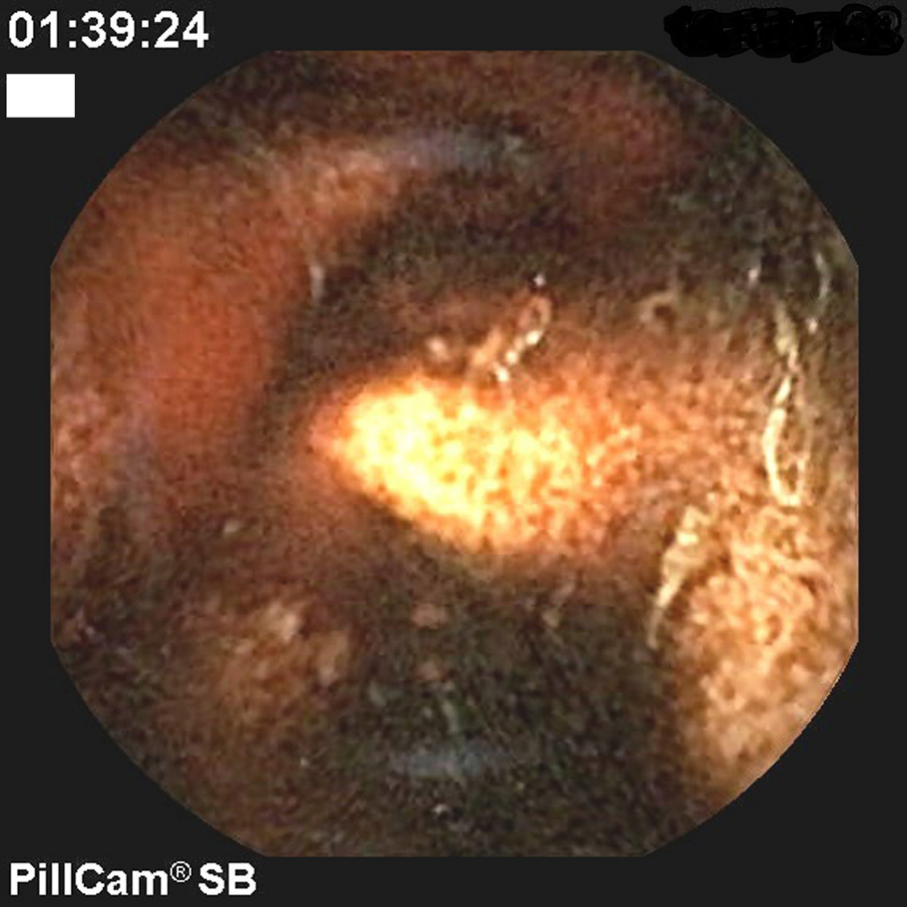
**Portal hypertensive enteropathy. Black stools in the jejunum with areas of spontaneous active bleeding**.

An 83-year-old Caucasian woman with cirrhosis was admitted several times to our hospital for anemia and positive fecal occult blood tests. The medical history included cirrhosis-related hepatitis C virus (HCV) diagnosed 18 years ago and a recent diagnosis of atrial flutter/fibrillation treated only with β-blockers drugs (neither anticoagulant nor antiplatelet therapy was prescribed because of the anemia). Four years ago, she presented with ascites that was treated with paracentesis and diuretic drugs. No other episodes of ascites were reported in the follow-up, but anemia was constantly present, and the patient underwent multiple blood transfusions. In two years, upper gastrointestinal (GI) endoscopy was performed three times, and this revealed esophageal varices (F2, according to NIEC classification [10]) without signs of bleeding. There was severe hypertensive gastropathy, but active bleeding or clots in the stomach were never observed. Colonoscopy was negative. We started therapy with β-blockers (40 mg/day at maximum dosage because of hypotension) first, and later with octreotide, without any significant effect on the anemia (mean hemoglobin level, 6 to 7 g/dl). To rule out any other cause of anemia, we performed a CE that showed severe portal hypertensive gastropathy (Figure 1) and diffuse erythema of the jejunum mucosa with melena and active oozing bleeding (Figure 2). Because of the persistent anemia that required frequent blood transfusions, we decided on a TIPS placement. During the procedure, the portosystemic pressure gradient was measured, showing a value of 13 mm Hg, After the TIPS, no other anemia or bleeding episodes were observed in the follow-up, and the hemoglobin level increased to 9 to 10 g/dl. Two months later, the patient was readmitted to our hospital for portal encephalopathy and jaundice. The initial laboratory results revealed the following: hemoglobin, 9.5 g/dl; hematocrit, 31%; serum urea nitrogen, 42 mmol/L; PT, 2.2INR; PLT. 70,000/mm^3^; bilirubin, 45 mg/dl (total, 30 mg/dl); blood ammonia level, 125 mg/dl.

**Figure 3:**
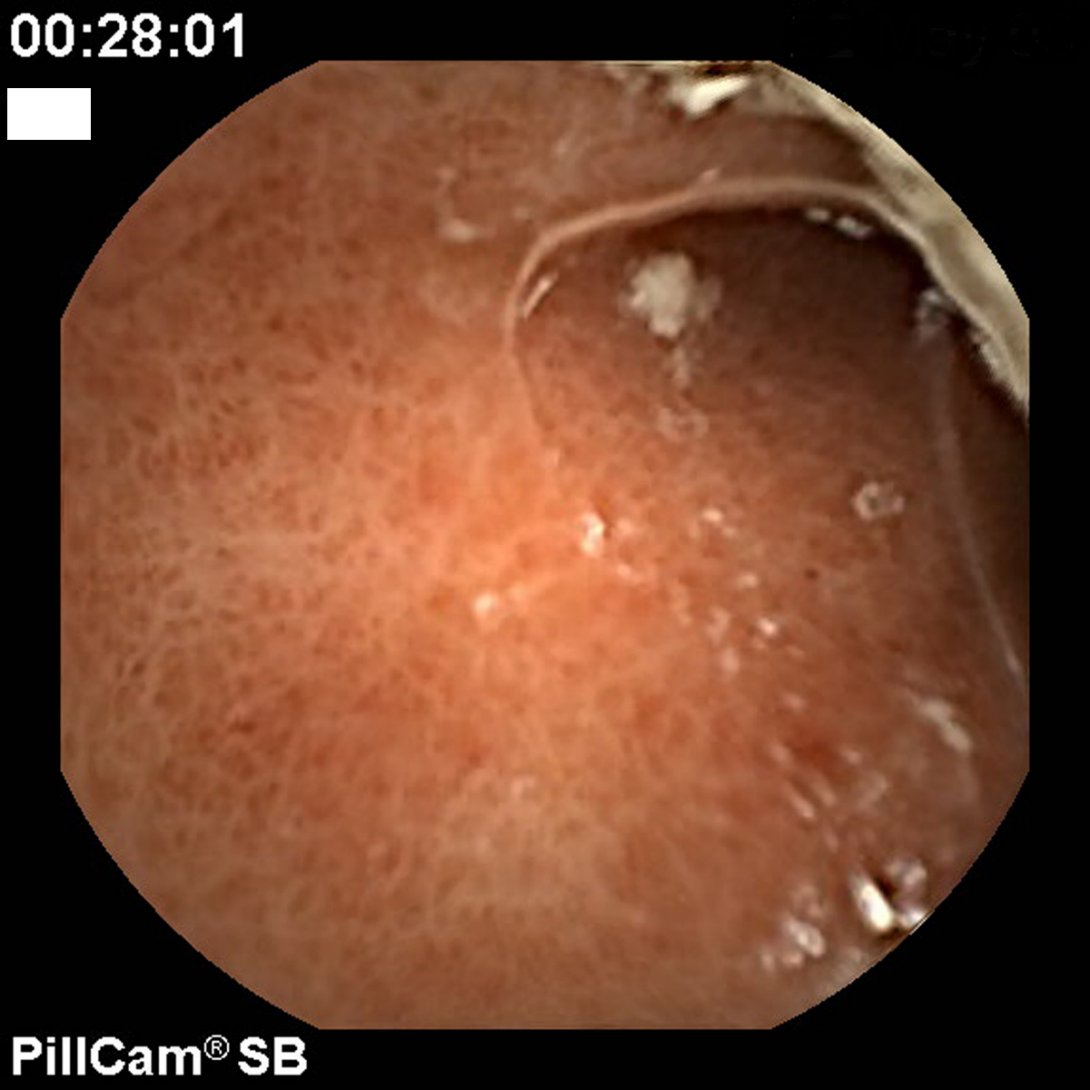
**Gastric pictures after transjugular intrahepatic portosystemic shunt (TIPS) placement. Mosaic pattern of the antrum without any bleeding signs**.

**Figure 4:**
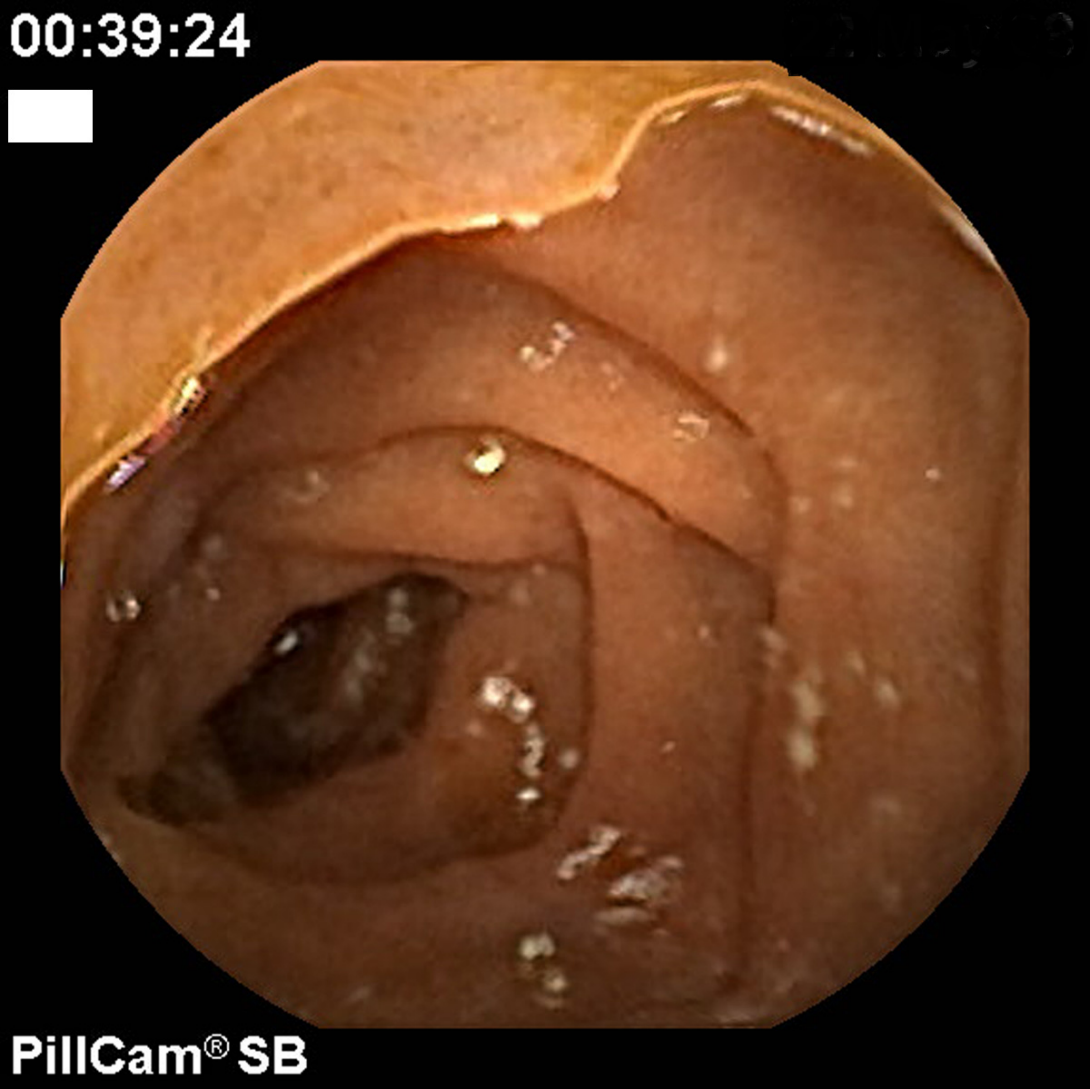
**Small bowel mucosa after transjugular intrahepatic portosystemic shunt (TIPS). Jejunal mucosa with minimal cherry-red spots without any bleeding signs**.

We started therapy with lactulose, antibiotics, and fluids; moreover, we repeated the CE, which showed a dramatic improvement in the gastric (Figure 3) and jejunal picture without evidence of bleeding (Figure 4). The patient died 15 days later of liver failure, but the hemoglobin level remained stable for that time.

## Discussion

In this case report, we describe the changes that occurred in the gastric and jejunal mucosa in a cirrhosis patient with portal hypertension with CE, and we report the positive effect of a TIPS placement. The clinical value and prevalence of PHE are not well known, but with the advent of CE, this entity seems more common, and some authors report a prevalence of more than 60% [6-9].

These data confirm that the hemodynamic alteration of portal hypertension can produce relevant effects in the small bowel mucosa, similar to those found in the gastric mucosa.

De Palma *et al. *[6] and Goulas *et al. *[7] observed that the frequency of the PHE increases with worsening of Child-Pugh class, but other authors have not found this correlation [8,9]. Moreover, active ileal bleeding has been observed in 5% to 10% of cirrhosis patients [6,9]. Our patient had anemia as the main symptom; she had severe portal hypertension, although no variceal bleeding was observed, and the global liver function was satisfactory. In our patient, the anemia was due both to portal hypertensive gastropathy and to active bleeding observed in the jejunum by CE. The correlation between portal hypertensive gastropathy and PHE was also observed in a previous study [7].

A multicenter study, published in abstract form, reported the results of CE in cirrhosis patients with unexplained bleeding [11], stressing the usefulness of CE results in the management of patients with portal hypertension. Our patient was treated with β-blockers and octreotide, without any significant effect. A number of pharmacologic agents have been used in acute bleeding due to portal hypertensive gastropathy; nonselective β-blockers and octreotide have been found to be successful in a high percentage of cases, whereas vasopressin and omeprazole were not so effective. Few data are available regarding the management of chronic bleeding, and the use of β-blockers has shown minimal improvement in such patients [12]. The effect of drug therapy on PHE was reported in anecdotal cases only. Surgical shunt is another option in patients with severe portal hypertension, but we did not consider it for this patient because of the high operative risk and multiple co-morbidities. In our case, the CE finding has prompted us to consider TIPS placement to control bleeding. TIPS is a well-known technique to reduce portal hypertension and to control variceal bleeding [13], but no data are available about its effect on jejunal mucosa in cases of PHE. The American Association for the Study of Liver Disease (AASLD) suggests TIPS as the preferred approach for prevention of recurrent bleeding in patients with ectopic varices [14]. Our patient showed an improvement of anemia after TIPS, and we were able to show the beneficial effect of TIPS placement on mucosal damage. Unfortunately, the patient died about three months later but we know that the one-year survival after TIPS varies from 48% to 90%, according to the severity of the liver disease [15]. Nonetheless, this case shows that the decompression of the portal vein after TIPS placement had a positive effect, not only on esophageal varices and hypertensive gastropathy, but also on the duodenal-jejunal mucosa. To our knowledge, this is the first description of a significant improvement of PHE after TIPS placement documented by CE.

## Conclusions

CE has made it possible to examine the small bowel mucosa directly and has allowed endoscopists to visualize the mucosal changes in cirrhosis patients with portal hypertension. This case underlines the fact that hemodynamic alterations of portal hypertension can produce relevant effects on the small-bowel mucosa. Therefore, we must bear in mind that the jejunum can also be a source of bleeding, and in cirrhosis patients with unexplained anemia, CE should be considered to optimize the treatment of portal hypertension.

## Competing interests

The authors declare that they have no competing interests.

## Consent

Written informed consent was obtained from the next-of-kin for publication of this manuscript and accompanying images. A copy of the written consent is available for review by the Editor-in-Chief of this journal

## Authors' contributions

AP made substantial contributions to the conception and drafting the article. He performed the capsule endoscopy procedure. NF monitored the patient during the admission periods. LS followed up the patient during the admission periods. AZ performed the ultrasound examination of the patient before and after TIPS placement. VC performed several upper GI endoscopies in the patient. AC helped in analysis and interpretation of the data. SG made suggestions in writing the manuscript and the final approval of the article. All authors read and approved the final manuscript
